# Supplementary material for: Synthesis of Amino Acid–Naphthoquinones and In Vitro Studies on Cervical and Breast Cell Lines
Source: Molecules. 2019 Nov 25;24(23):4285. doi: 10.3390/molecules24234285 (PMC6930466; doi:10.3390/molecules24234285)

Supplementary data

Synthesis of amino acid-naphthoquinones and *in vitro* studies on cervical and breast cell lines

Ernesto Rivera-Avalos [1], Denisse de Loera [1], Jorge G. Araujo-Huitrado [2], Ismailia L. Escalante-García [3], Miguel A. Muñoz-Sánchez [3], Hiram Hernández [3], Jesús A. López [2,*] and Lluvia López [4,*]

[1] School of Chemistry, Universidad Autónoma de San Luis Potosí, Av. Manuel Nava No. 6, SLP 78210, México; neto_riava@hotmail.com (E.R.-A.); atenea.deloera@uaslp.mx (D.d.L.)

[2] Laboratorio de microRNAs y Cáncer, Unidad Académica de Ciencias Biológicas, Universidad Autónoma de Zacatecas, Av. Preparatoria No.301, ZAC 98068, México; jalopez2210@gmail.com (J.G.A.-H.); jalopez@uaz.edu.mx (J.A.L.)

[3] Unidad Académica de Ciencias Químicas, Universidad Autónoma de Zacatecas, Carr. Zacatecas – Guadalajara km 6, ZAC 98160, México; ileg@uaz.edu.mx (I.L.E.-G.); 27804270@uaz.edu.mx (M.A.M.-S.); hiram.hernandez.lopez@uaz.edu.mx (H.H.)

[4] Instituto de Investigación de Zonas Desérticas, Universidad Autónoma de San Luis Potosí, Altaír No. 200, SLP 78377, México; lluvia.lopez@uaslp.mx (L.L.)

* Correspondence: lluvia.lopez@uaslp.mx (L.L.); jalopez@uaz.edu.mx (J.A.L.); Tel.: +52-444-826-2300 (L.L.); +52-492-149-2648 (J.A.L.)

CONTENT

1. Infrared characterization 2
   Amino acids-1,4-naphthoquinone derivatives: 2
   Amino acids-2,3-dichloronaphthoquinone derivatives: 4
2. NMR characterization. 7
   Amino acids-1,4-naphthoquinone derivatives: 7
   Amino acids-2,3-dichloronaphthoquinone derivatives: 10
3. Mass spectrometry characterization of compounds 3c, 3e, 4b, 4c and 4e. 14

1. Infrared characterization

Amino acids-1,4-naphthoquinone derivatives:

(3a)

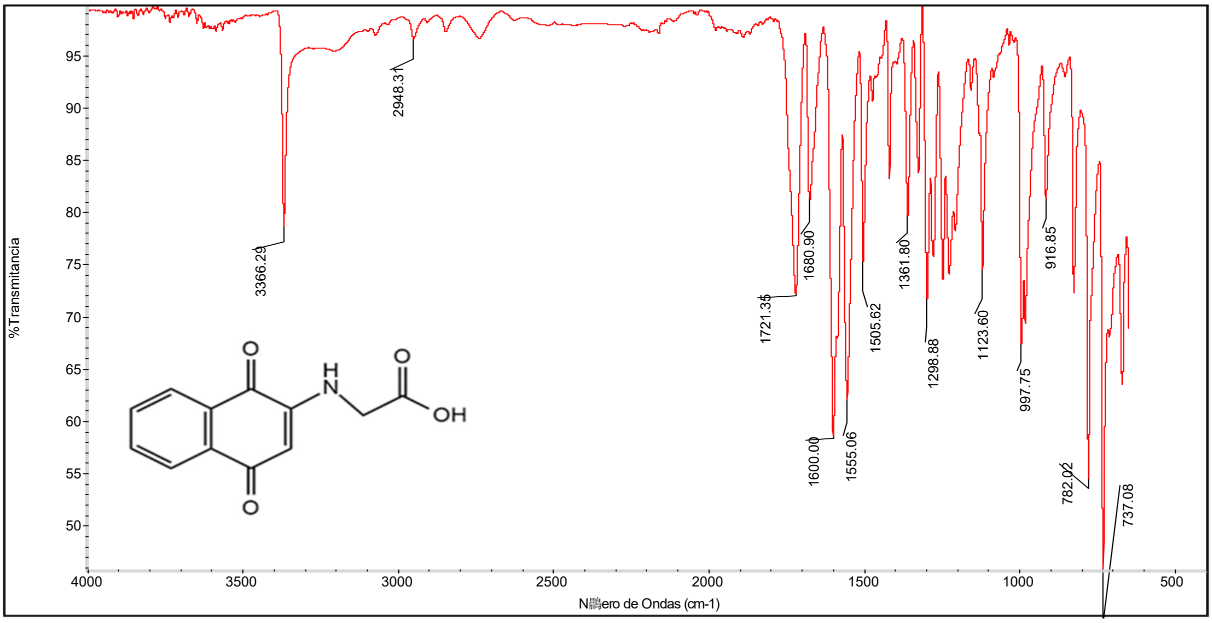

(3b)

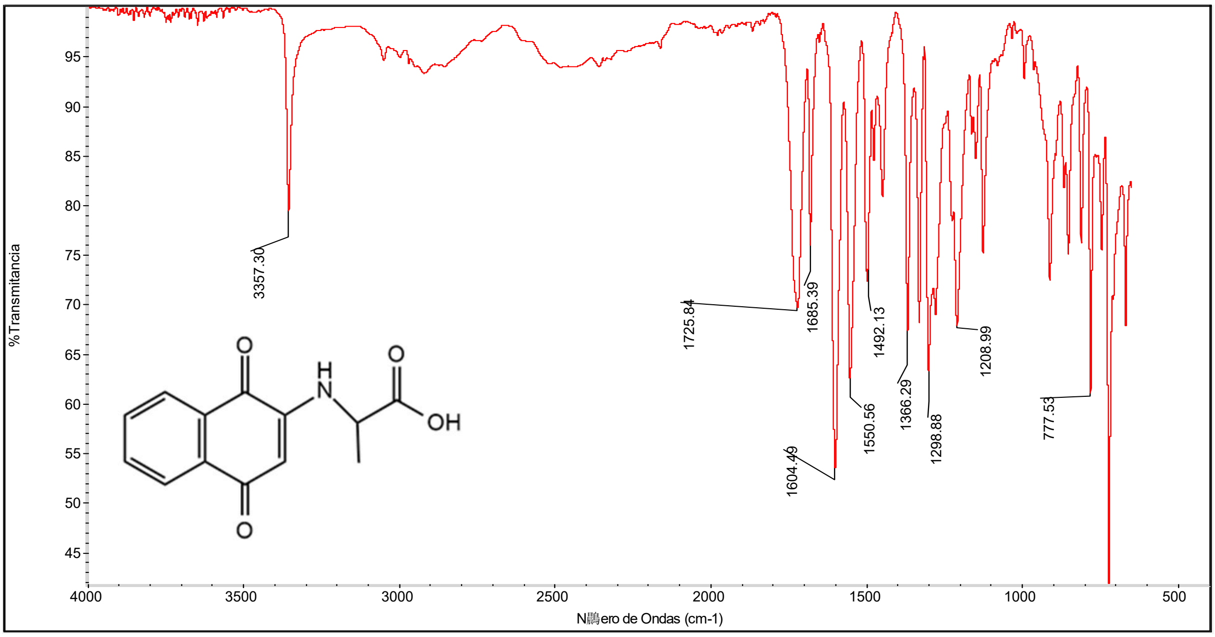

(3c)

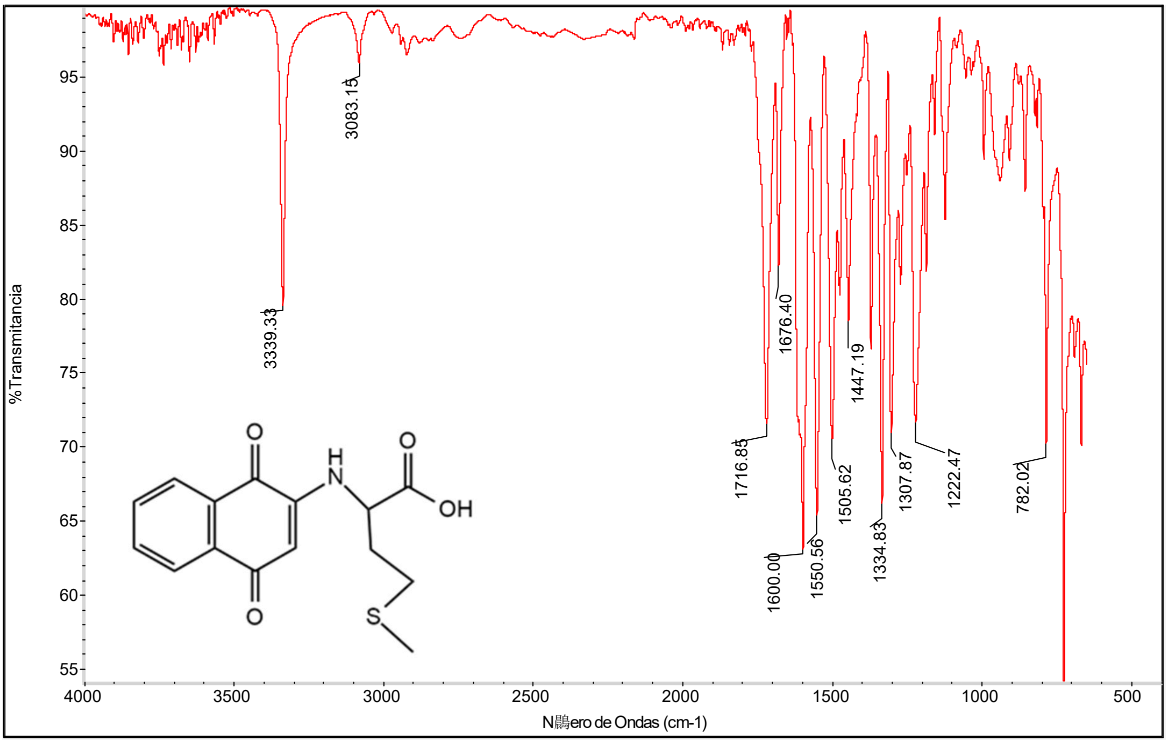

%Transmitancia
N鵬ero de Ondas (cm-1)
3339.33
3083.15
1716.85
1676.40
1600.00
1550.56
1505.62
1447.19
1334.83
1307.87
1222.47
782.02
O
H
N
OH
S

(3d)

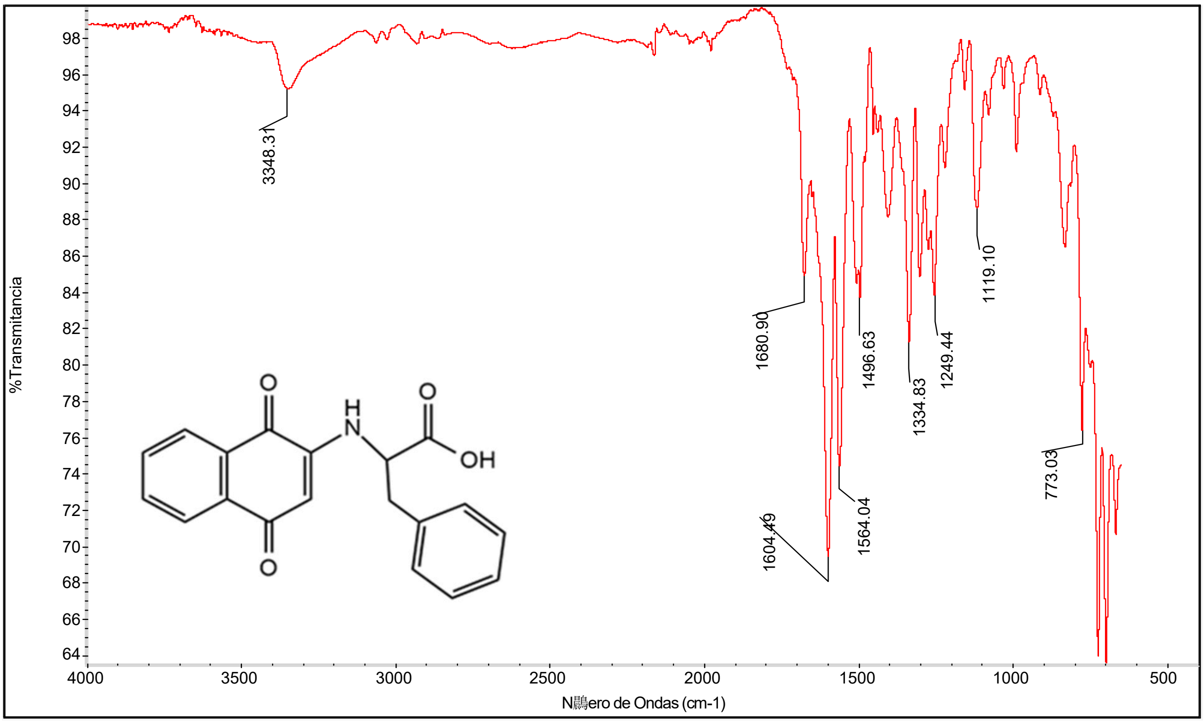

%Transmitancia
N鵬ero de Ondas (cm-1)
3348.31
1680.90
1604.49
1564.04
1496.63
1334.83
1249.44
1119.10
773.03
O
H
N
OH

(3e)

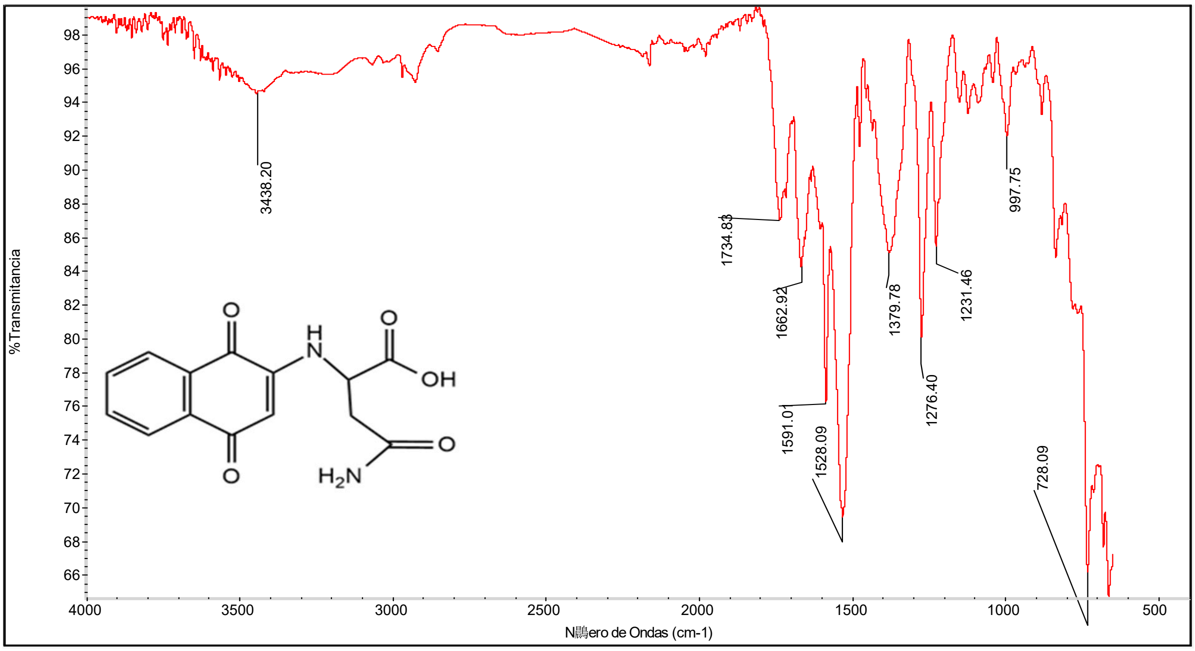

Amino acids-2,3-dichloronaphthoquinone derivatives:

(4a)

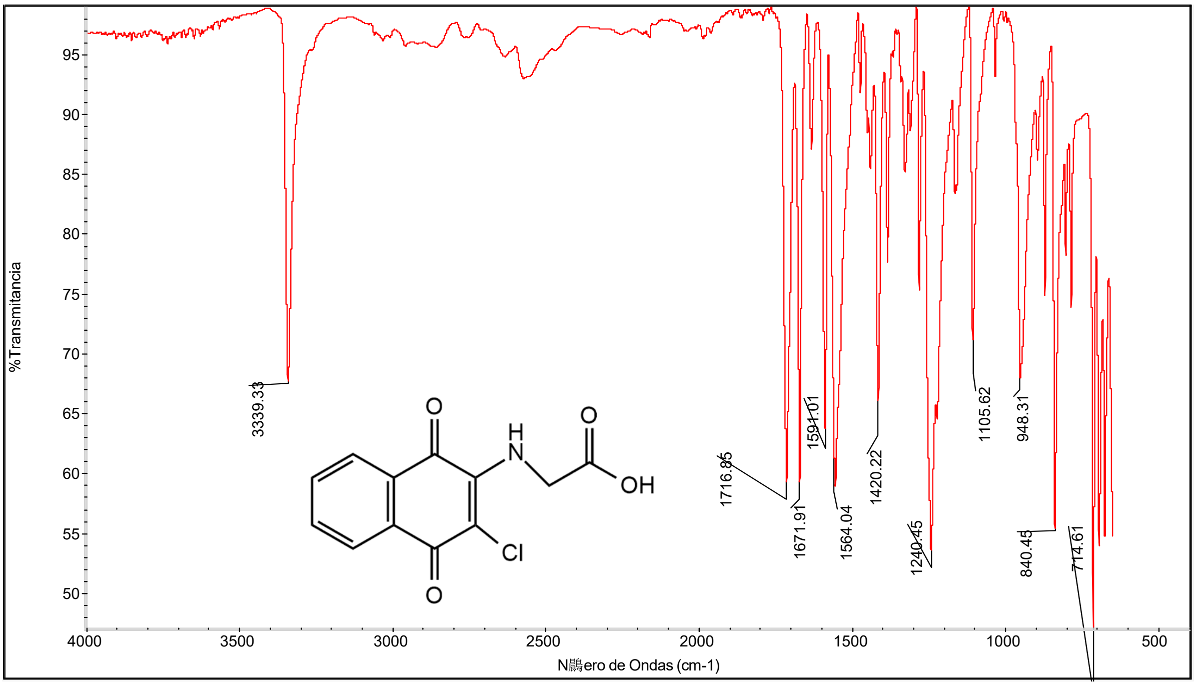

(4b)

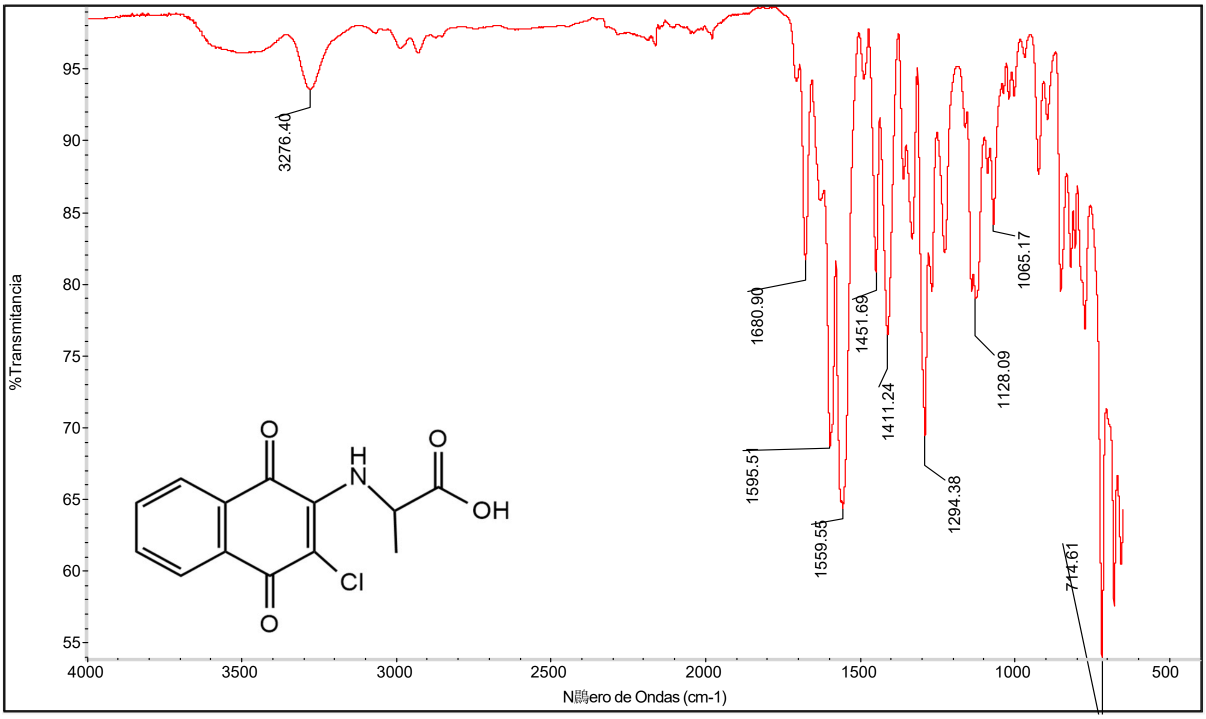
%Transmitancia
N鷗ero de Ondas (cm-1)
3276.40
1680.90
1595.51
1559.55
1451.69
1411.24
1294.38
1128.09
1065.17
714.61
O
H
N
O
OH
Cl
O

(4c)

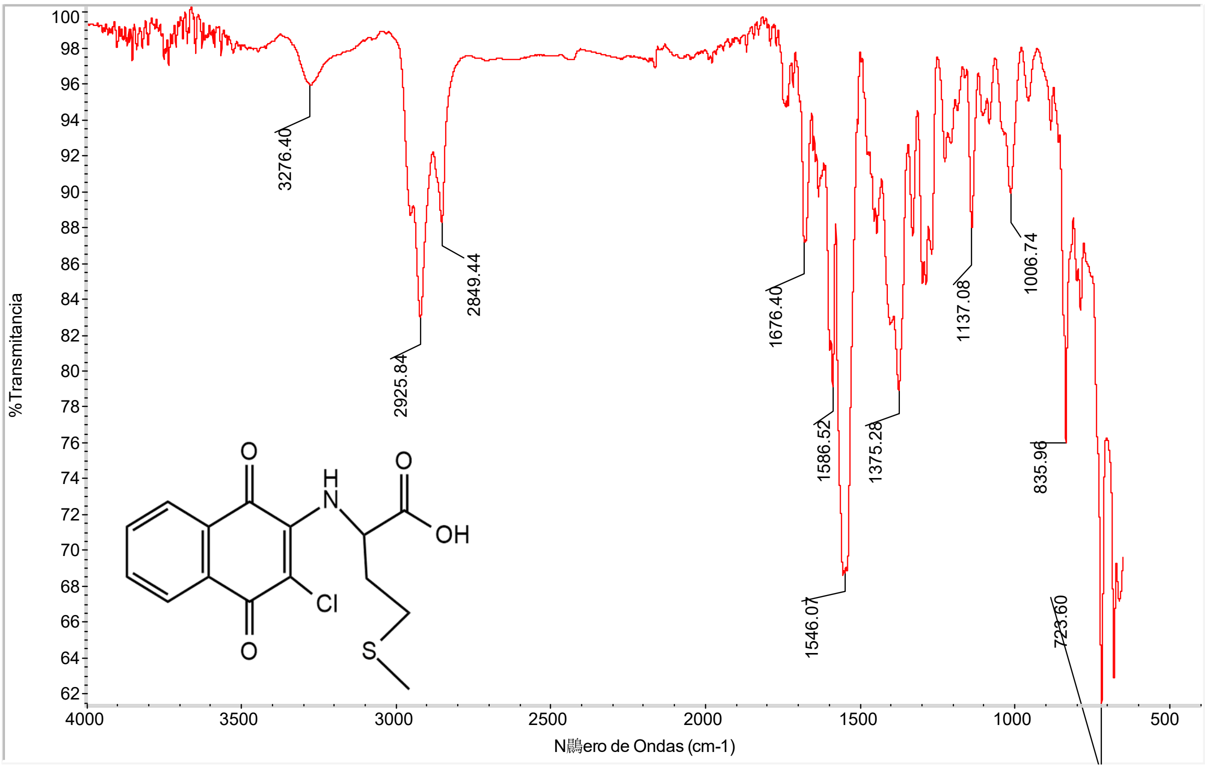
%Transmitancia
N鷗ero de Ondas (cm-1)
3276.40
2925.84
2849.44
1676.40
1586.52
1546.07
1375.28
1137.08
1006.74
835.96
723.60
O
H
N
O
OH
Cl
O
S

(4d)

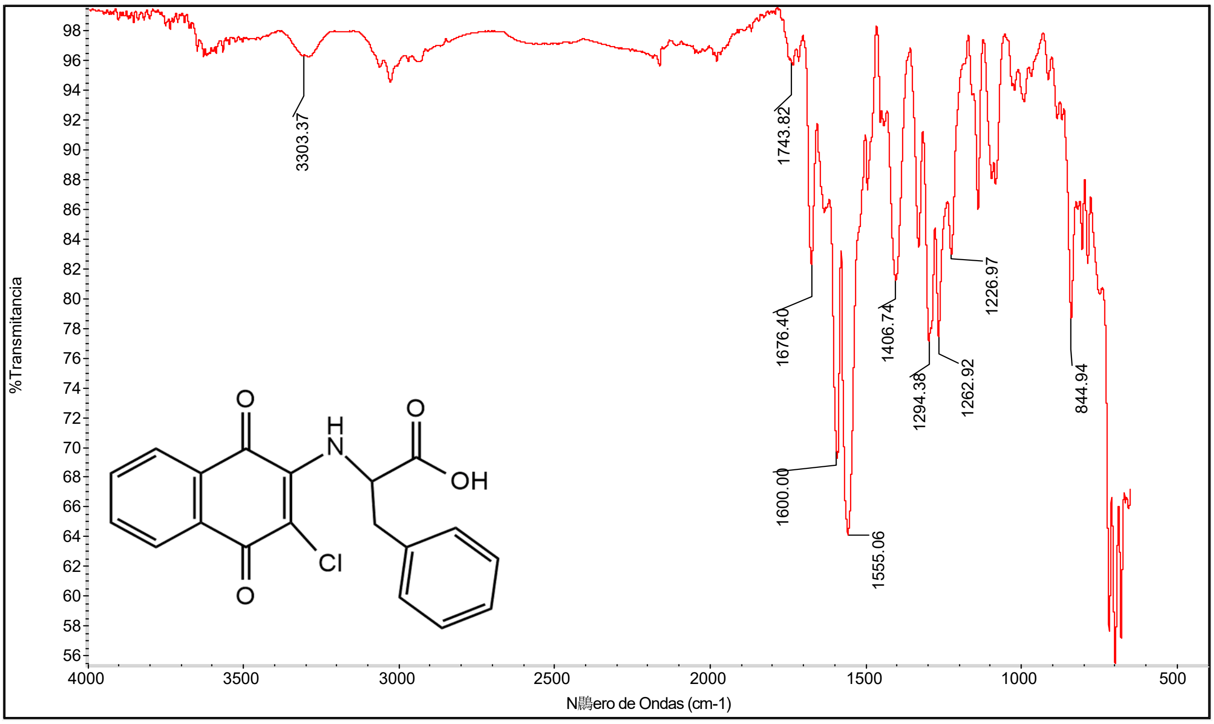
3303.37
1743.82
1676.40
1600.00
1555.06
1406.74
1294.38
1262.92
1226.97
844.94
%Transmitancia
N鷴ero de Ondas (cm-1)
O
O
H
N
O
OH
Cl

(4e)

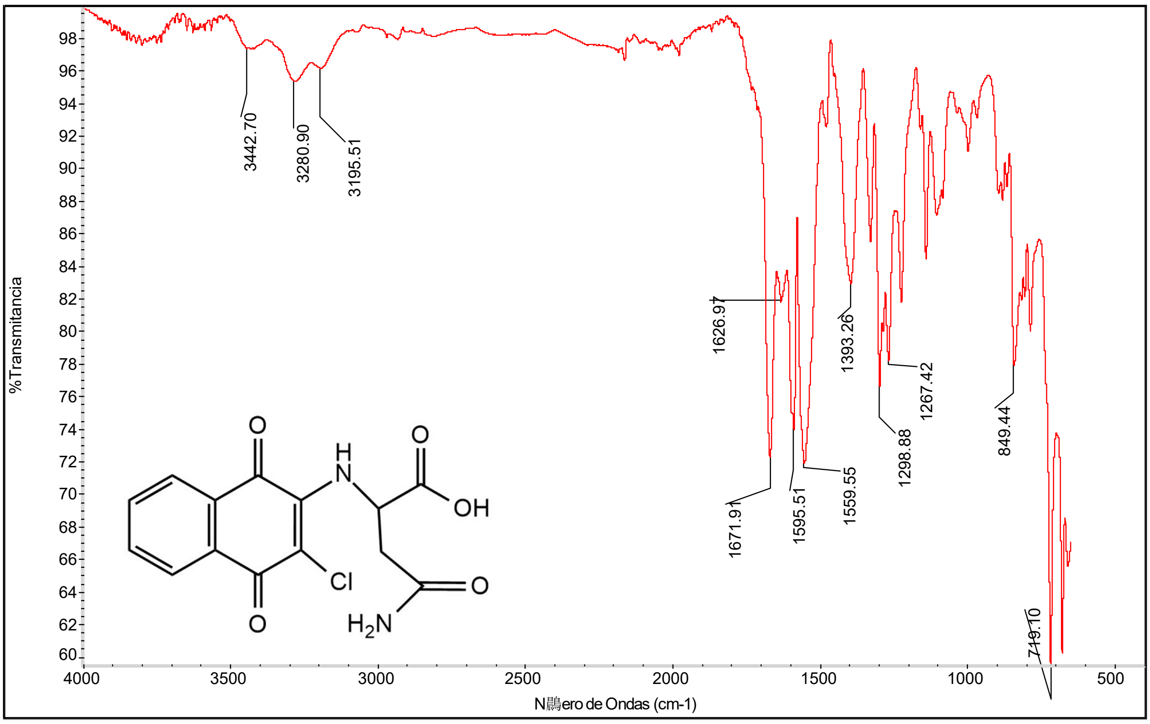
3442.70
3280.90
3195.51
1626.97
1671.91
1595.51
1559.55
1393.26
1298.88
1267.42
849.44
719.10
%Transmitancia
N鷴ero de Ondas (cm-1)
O
O
H
N
O
OH
Cl
H_2N
O

2. NMR characterization.

Amino acids-1,4-naphthoquinone derivatives:

(3a)

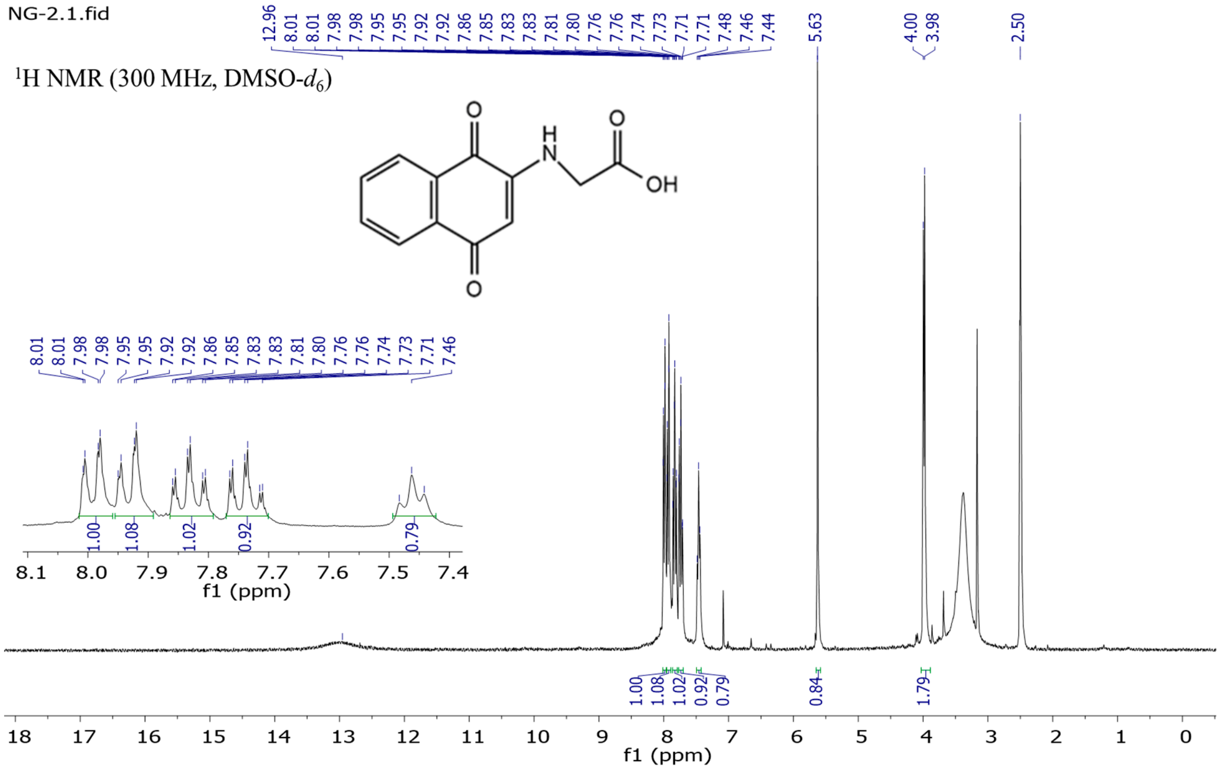

(3b)

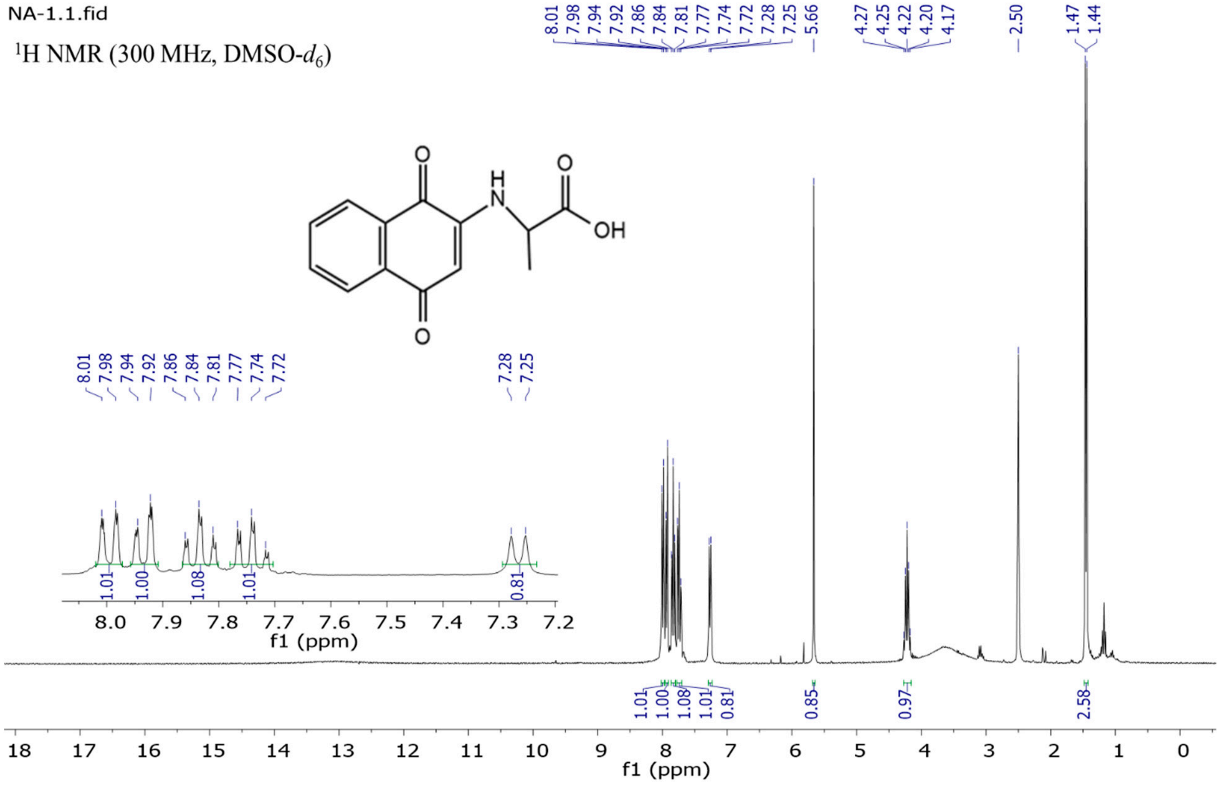

(3c)

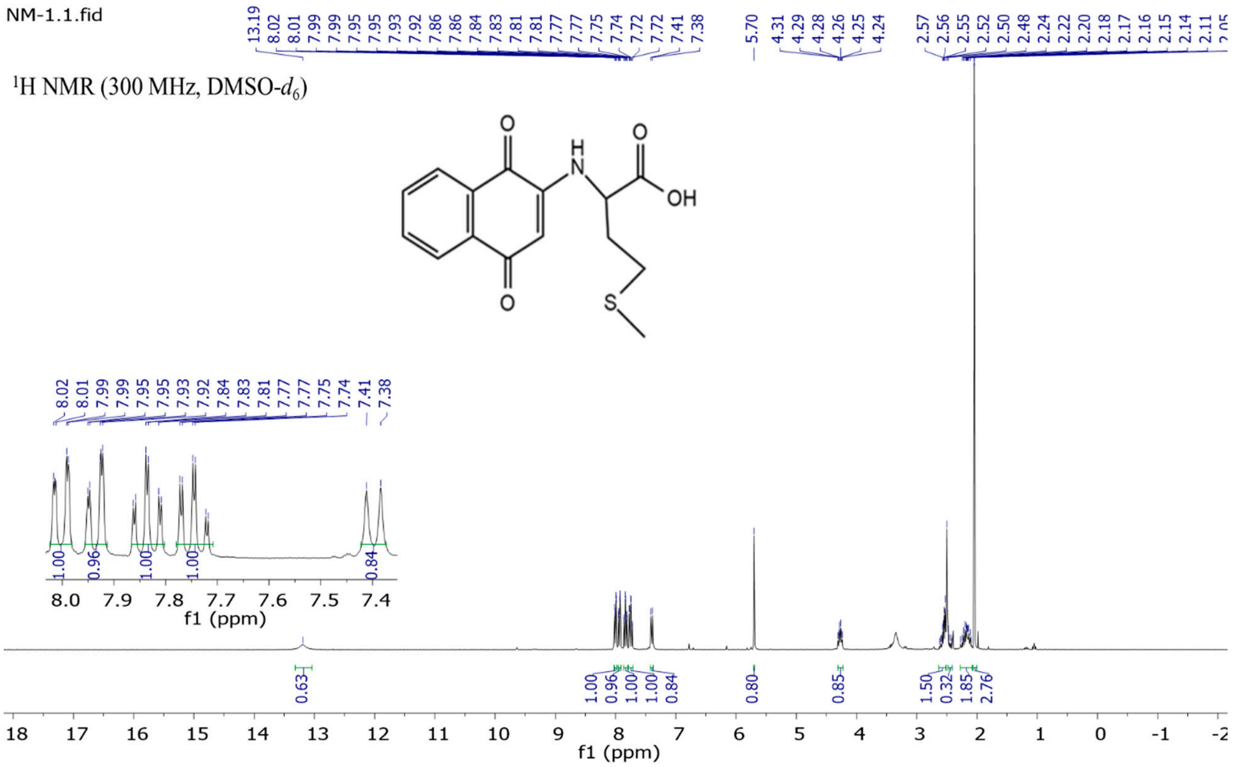
NM-1.1.fid
^{1}H NMR (300 MHz, DMSO-d_6)
13.19 8.02 8.01 7.99 7.99 7.95 7.95 7.93 7.92 7.86 7.86 7.84 7.83 7.81 7.81 7.77 7.77 7.75 7.74 7.72 7.72 7.41 7.38
5.70
4.31 4.29 4.28 4.26 4.25 4.24
2.57 2.56 2.55 2.52 2.50 2.48 2.24 2.22 2.20 2.18 2.17 2.16 2.15 2.14 2.11
0.63 1.00 0.96 1.00 1.00 0.84 0.80 0.85 1.50 0.32 1.85 2.76
f1 (ppm)

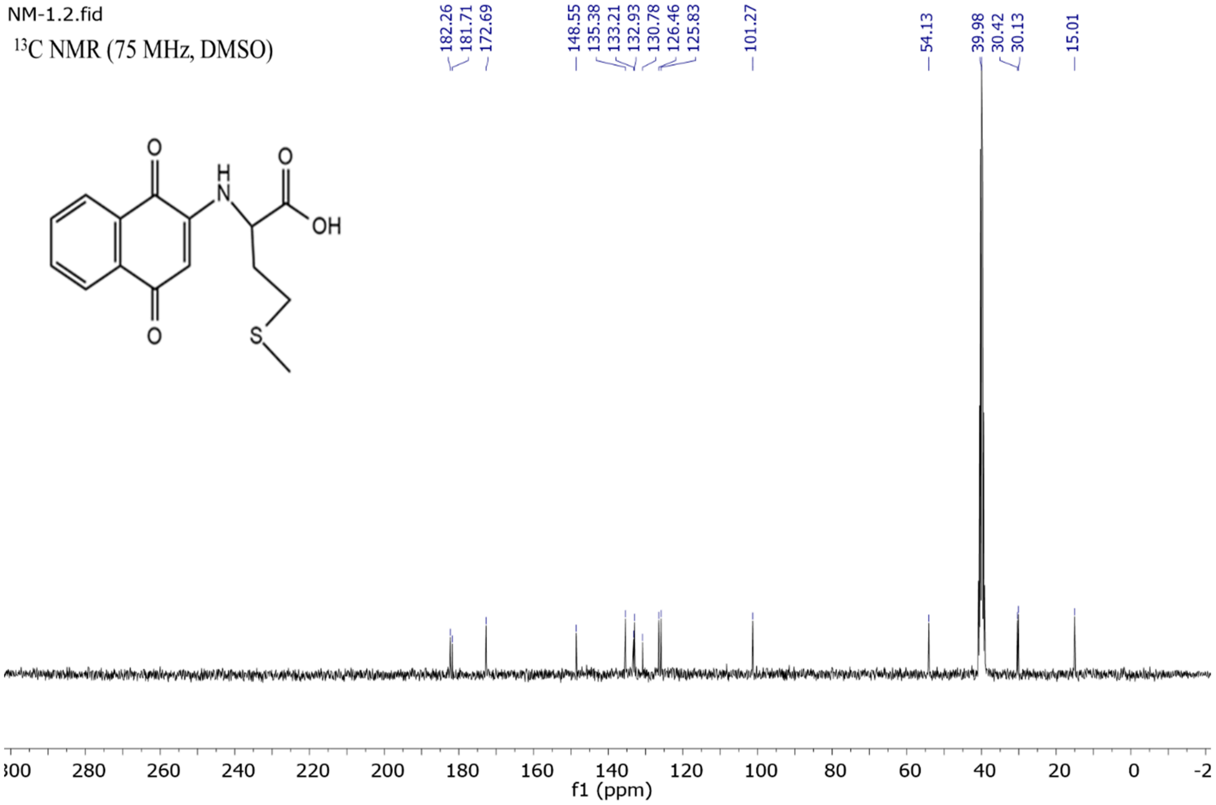
NM-1.2.fid
^{13}C NMR (75 MHz, DMSO)
182.26 181.71 172.69
148.55 135.38 133.21 132.93 130.78 126.46 125.83
101.27
54.13
39.98 30.42 30.13
15.01
f1 (ppm)

(3d)

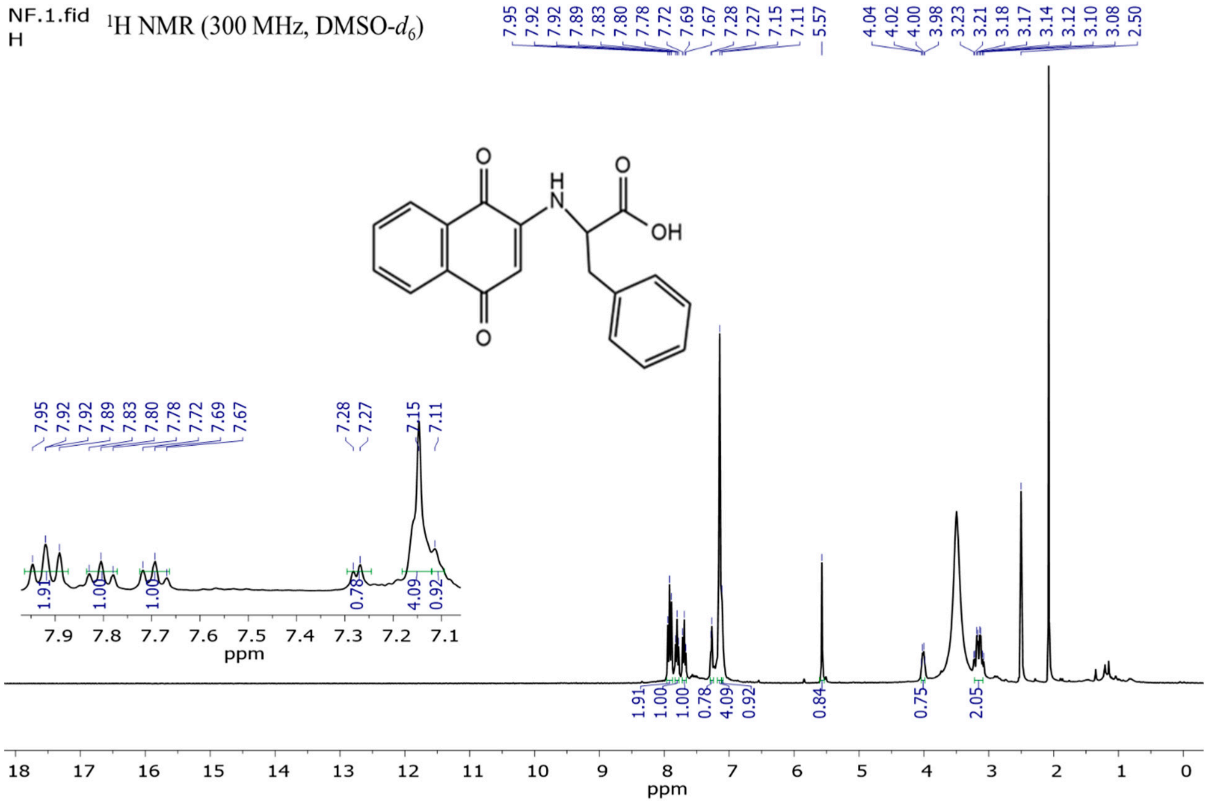
NF.1.fid
H
^{1}H NMR (300 MHz, DMSO-d_6)
7.95 7.92 7.92 7.89 7.83 7.80 7.78 7.72 7.69 7.67 7.28 7.27 7.15 7.11 5.57 4.04 4.02 4.00 3.98 3.23 3.21 3.18 3.17 3.14 3.12 3.10 3.08 2.50
O
H
N
O
OH
O
1.91 1.00 1.00 0.78 4.09 0.92
ppm
1.91 1.00 1.00 0.78 4.09 0.92 0.84 0.75 2.05
18 17 16 15 14 13 12 11 10 9 8 7 6 5 4 3 2 1 0
ppm

(3e)

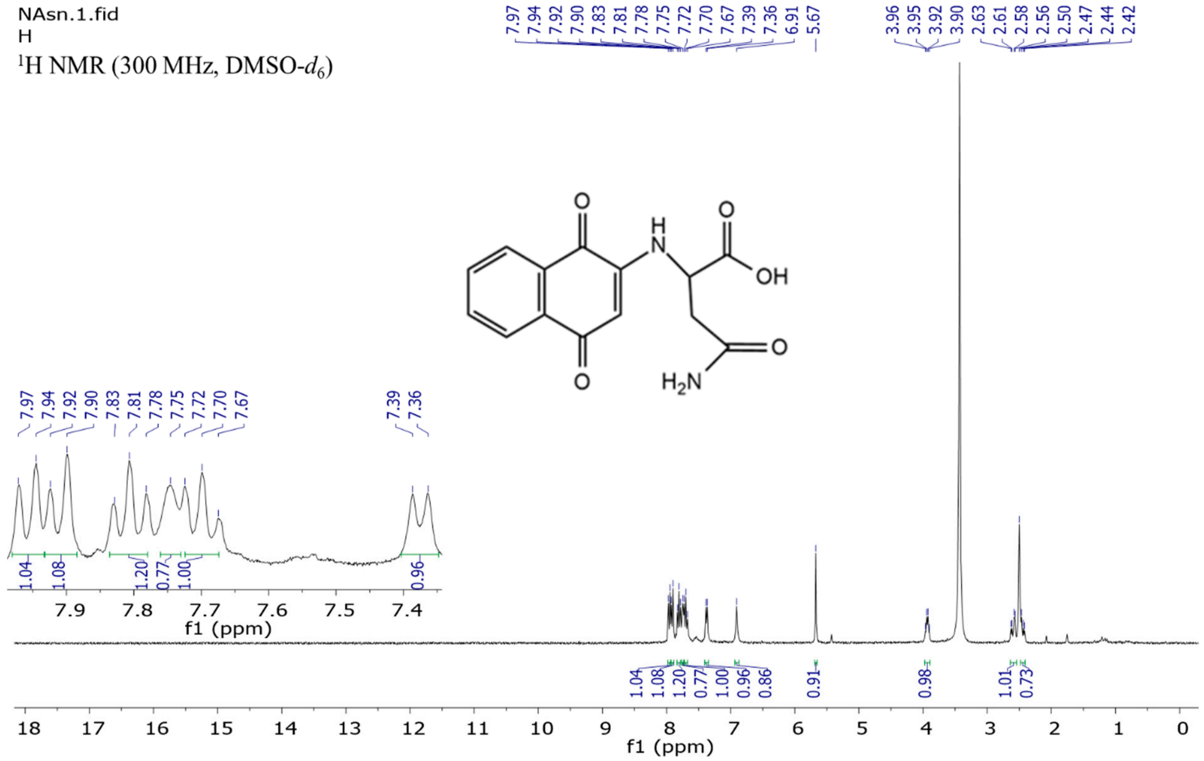
NAsn.1.fid
H
^{1}H NMR (300 MHz, DMSO-d_6)
7.97 7.94 7.92 7.90 7.83 7.81 7.78 7.75 7.72 7.70 7.67 7.39 7.36 6.91 5.67 3.96 3.95 3.92 3.90 2.63 2.61 2.58 2.56 2.50 2.47 2.44 2.42
O
H
N
O
OH
O
O
H_2N
1.04 1.08 1.20 0.77 1.00 0.96
f1 (ppm)
1.04 1.08 1.20 0.77 1.00 0.96 0.86 0.91 0.98 1.01 0.73
18 17 16 15 14 13 12 11 10 9 8 7 6 5 4 3 2 1 0
f1 (ppm)

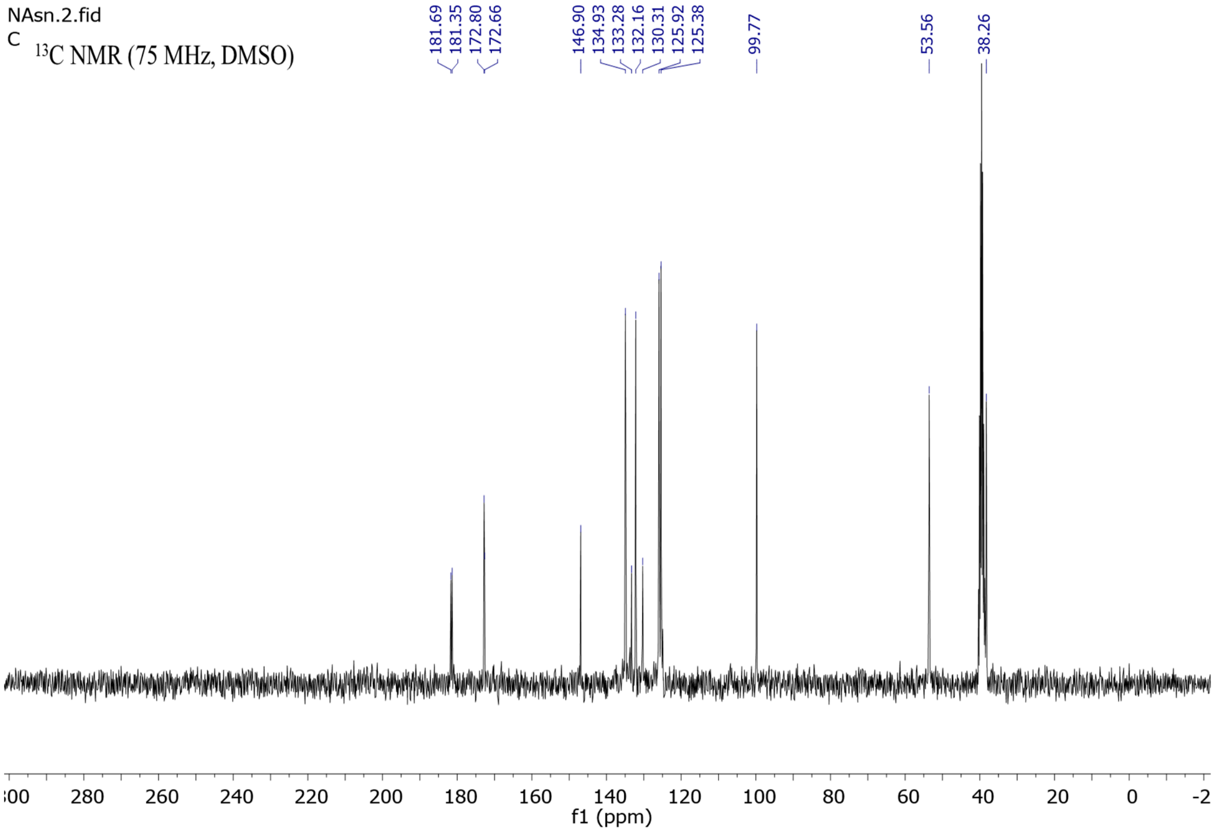

Amino acids-2,3-dichloronaphthoquinone derivatives:

(4a)

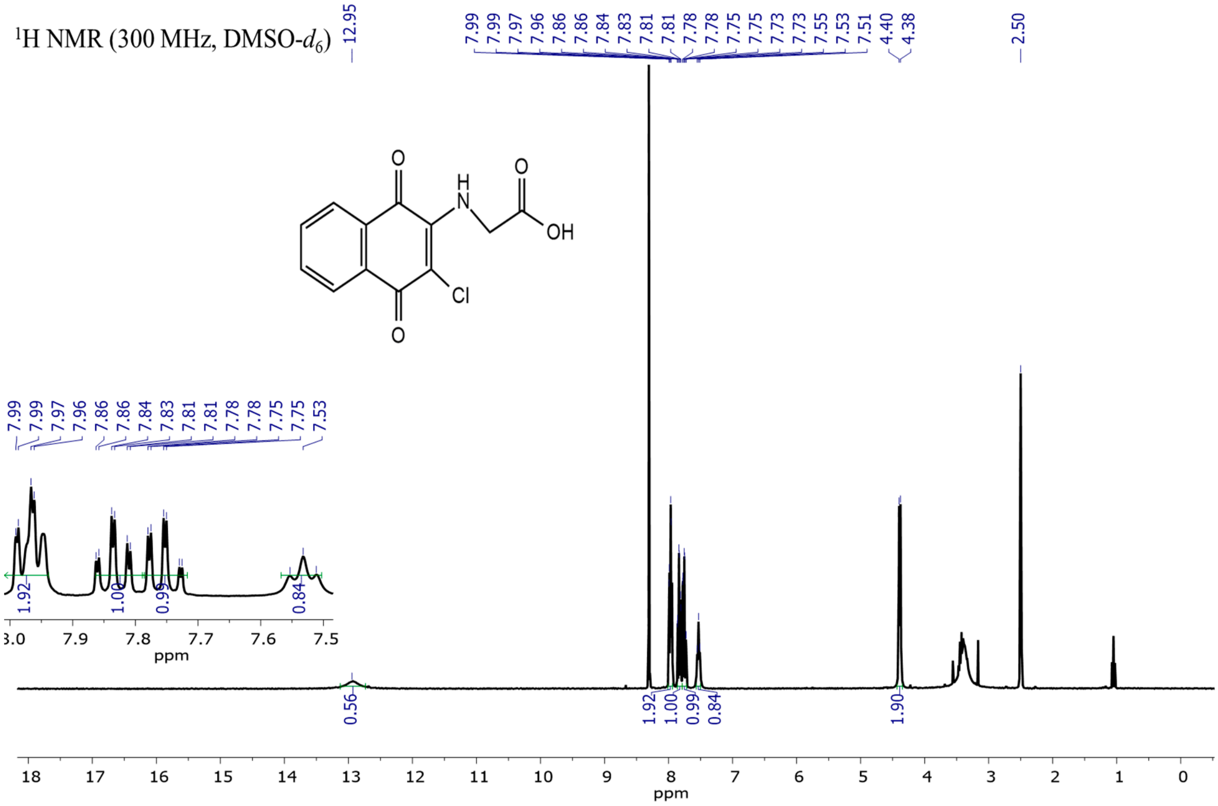

(4b)

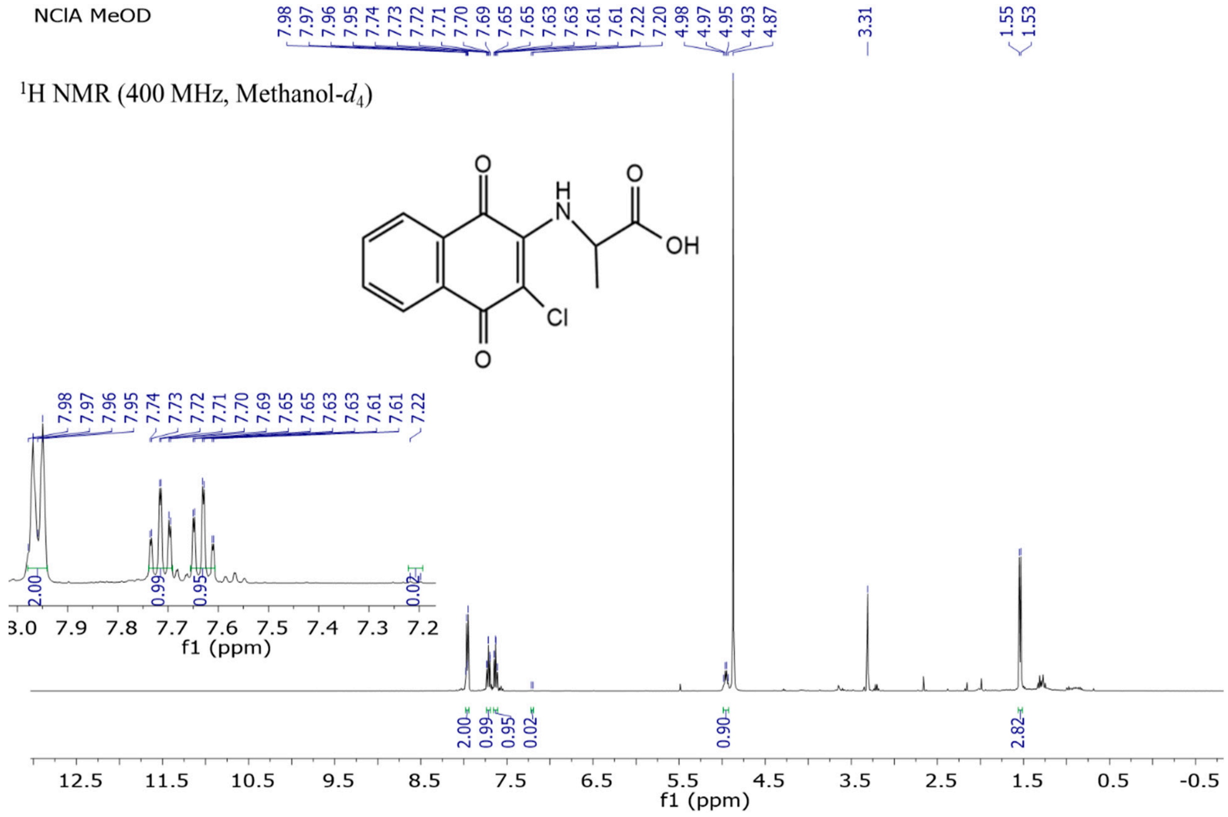
NClA MeOD
7.98 7.97 7.96 7.95 7.74 7.73 7.72 7.71 7.70 7.69 7.65 7.65 7.63 7.63 7.61 7.61 7.22 7.20 4.98 4.97 4.95 4.93 4.87
3.31
1.55 1.53
^{1}H NMR (400 MHz, Methanol-d_4)
2.00 0.99 0.95 0.02 0.90 2.82
f1 (ppm)

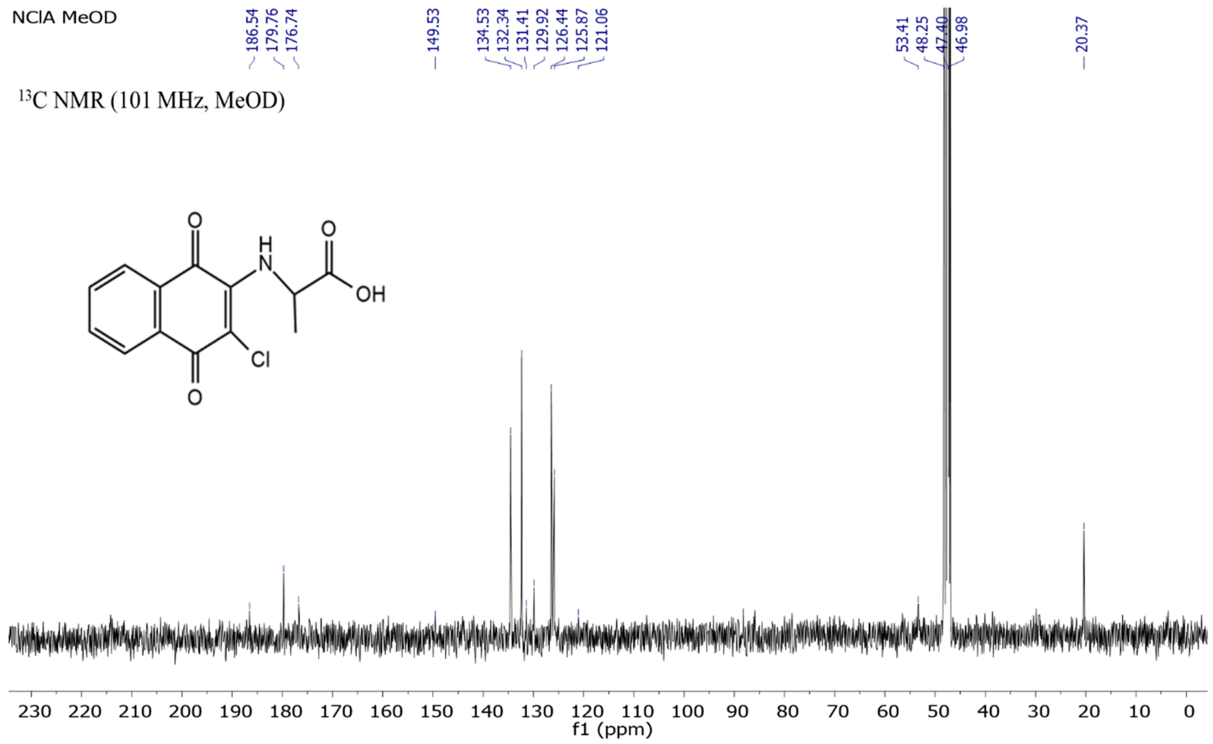
NClA MeOD
186.54 179.76 176.74
149.53
134.53 132.34 131.41 129.92 126.44 125.87 121.06
53.41 48.25 47.40 46.98
20.37
^{13}C NMR (101 MHz, MeOD)
f1 (ppm)

(4c)

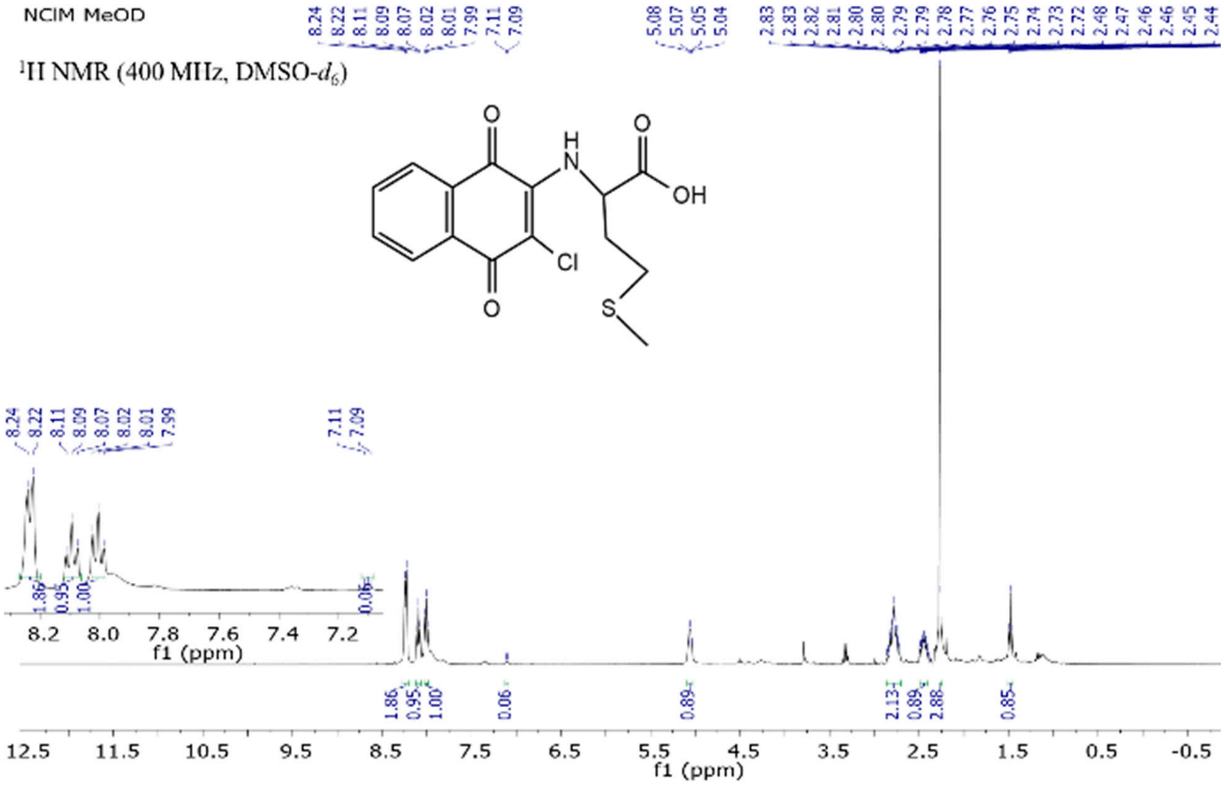

NCIM MeOD
¹H NMR (400 MHz, DMSO-d_6)
OH
Cl
f1 (ppm)

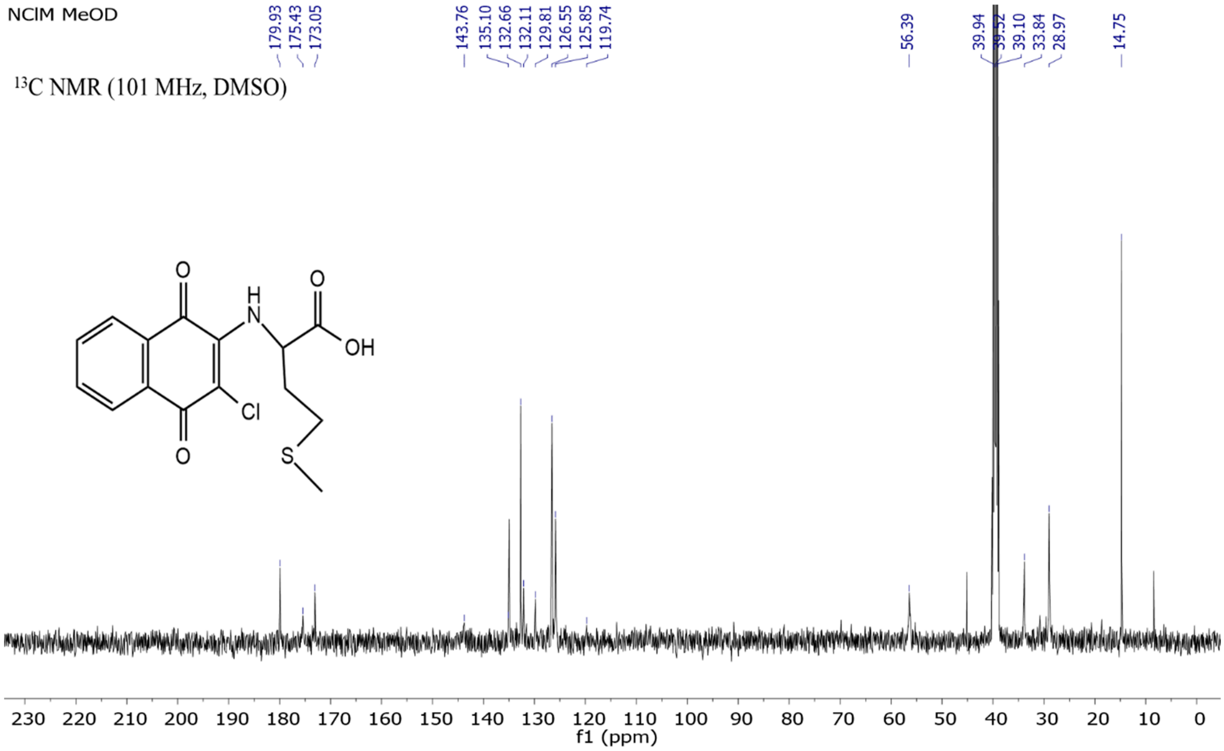

NCIM MeOD
¹³C NMR (101 MHz, DMSO)
OH
Cl
f1 (ppm)

(4d)

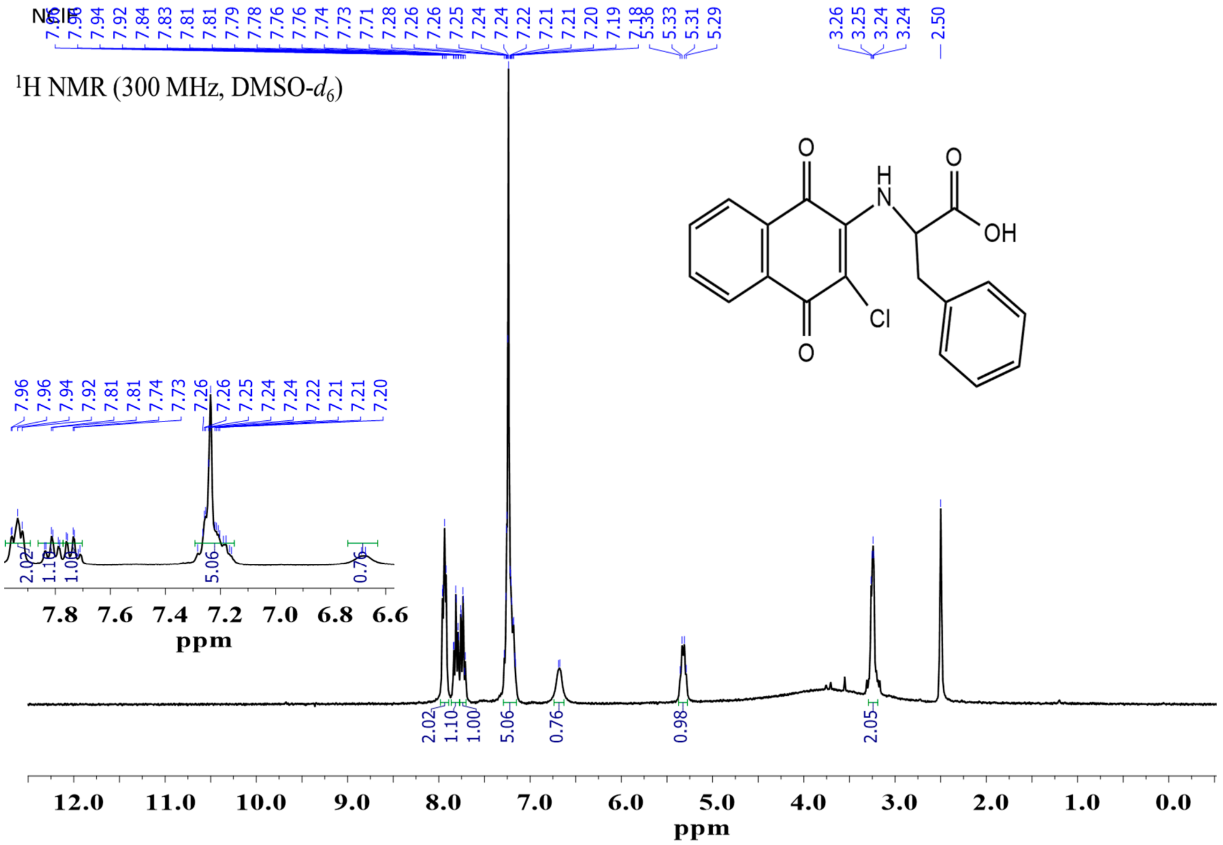

¹H NMR (300 MHz, DMSO-d6)
OH
Cl
ppm

(4e)

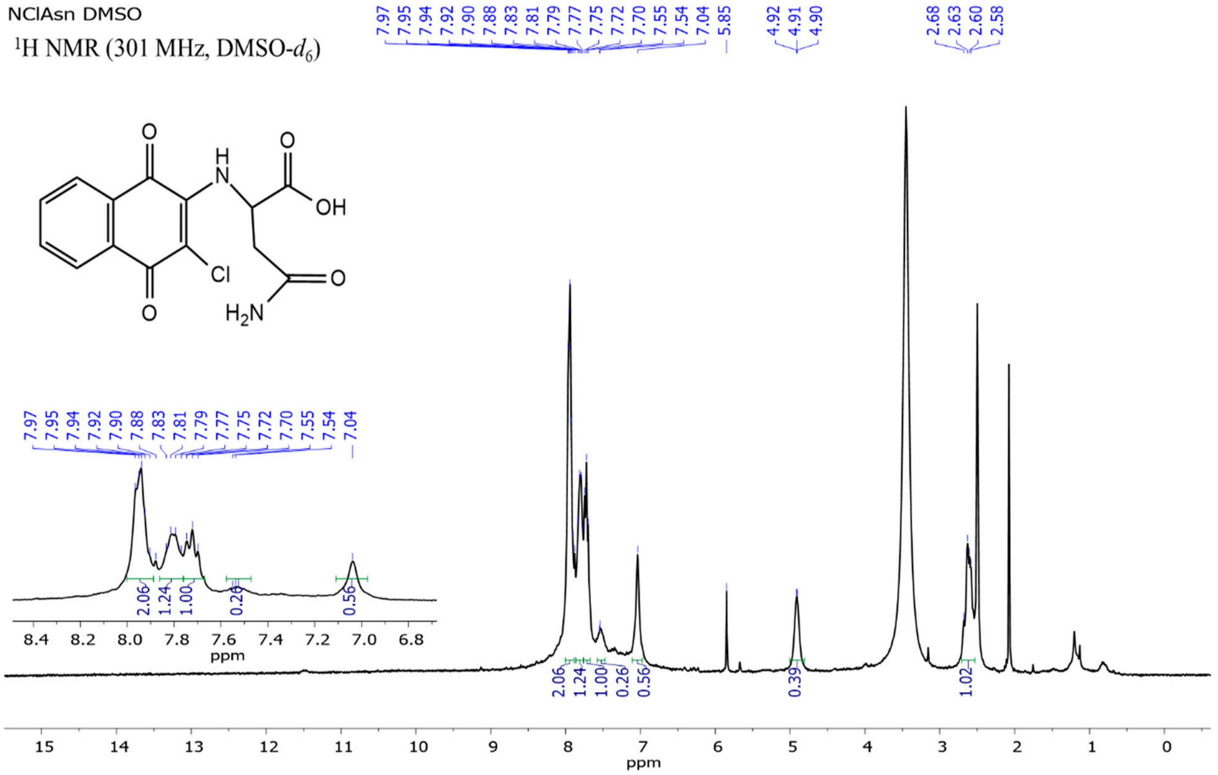

NClAsn DMSO
¹H NMR (301 MHz, DMSO-d6)
OH
Cl
H2N
ppm

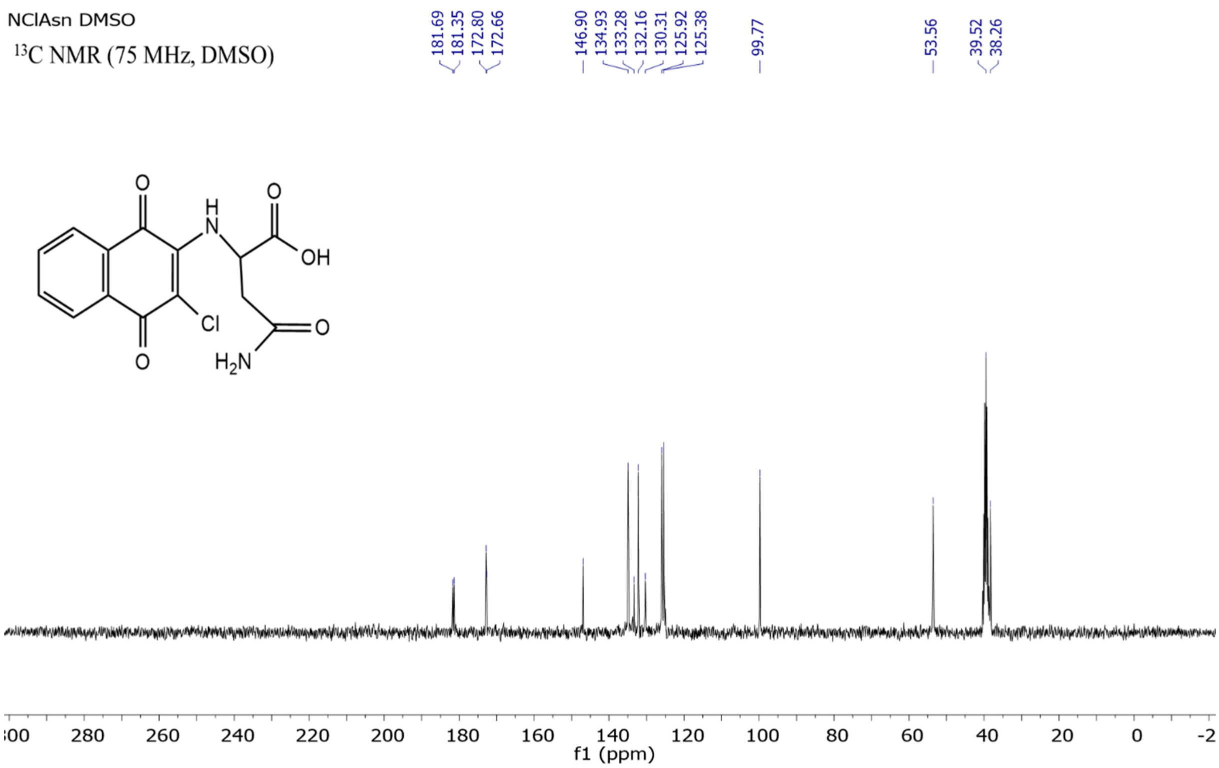

3. Mass spectrometry characterization of compounds 3c, 3e, 4b, 4c and 4e.

(3c)

Data:U-278 NM1-MS-GC
Sample Name:Lizbeth Rodriguez
Description:
Ionization Mode:ESI+
History:Determine m/z[Peak Detect[Centroid,30,Area];Correct Base[1.0%]];Correct Base[5.0%];Average(MS[1] 0..1)

Acquired:2/28/2018 10:26:25 AM
Operator:AccuTOF
Mass Calibration data:Cal Peg 600
Created:3/7/2018 9:26:00 AM
Created by:

Charge number:1 Tolerance:2.00(mmu) Unsaturation Number:0.0 .. 20.0 (Fraction:Both)
Element:^{12}C:0 .. 22, ^{1}H:1 .. 50, ^{14}N:0 .. 3, ^{16}O:3 .. 5, ^{32}S:1 .. 1

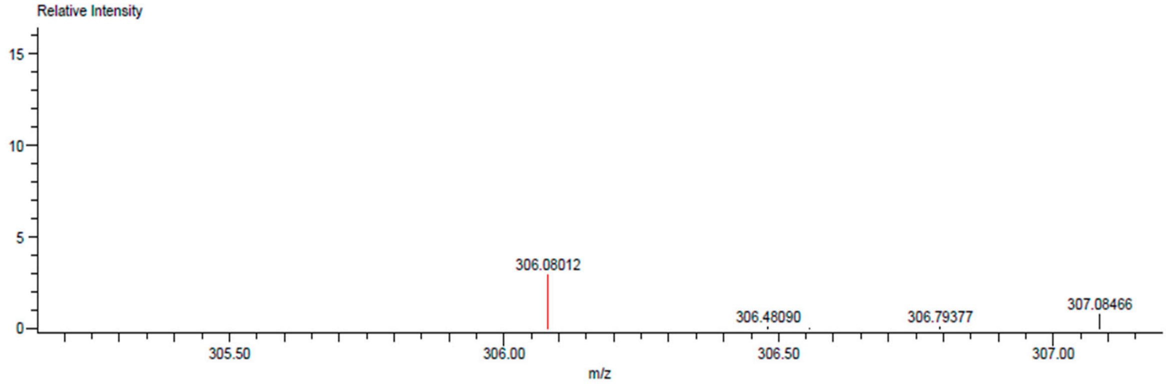

Mass	Intensity	Calc. Mass	Mass Difference (mmu)	Mass Difference (ppm)	Possible Formula	Unsaturation Number
306.08012	7591.35	306.08000	0.12	0.38	$^{12}C_{15}{}^{1}H_{16}{}^{14}N_{1}{}^{16}O_{4}{}^{32}S_{1}$	9.5

(3e)

**Data Filename**	NAsn.d	**Sample Name**	NAsn
**Sample Type**	Sample	**Position**	P1-B3
**Instrument Name**	LC QTOF-LANCIC	**User Name**	
**Acq Method**	Ine directa pos.m	**Acquired Time**	8/2/2018 2:19:53 PM
**IRM Calibration Status**	Success	**DA Method**	Default.m
**Comment**			

**Sample Group**		**Info.**	
**Stream Name**	LC 1	**Acquisition SW Version**	6200 series TOF/6500 series Q-TOF B.06.01 (B6172 SP1)

User Spectra

Fragmentor Voltage	Collision Energy	Ionization Mode
150	0	ESI

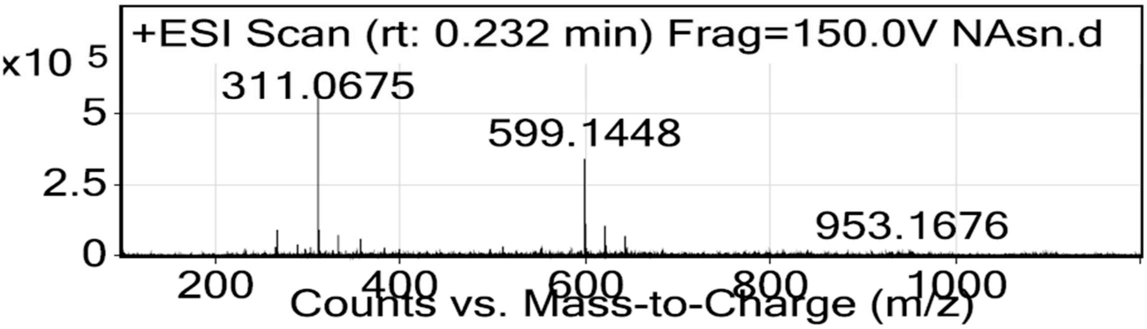

(4b)

Data:U-279 NCLA-MS
Sample Name:Lizbeth Rodriguez
Description:
Ionization Mode:ESI+
History:Determine m/z[Peak Detect[Centroid,30,Area];Correct Base[1.0%]];Correct Base[5.0%];Average(MS[1] 1..1)

Acquired:2/28/2018 10:29:53 AM
Operator:AccuTOF
Mass Calibration data:Cal Peg 600
Created:3/7/2018 9:35:13 AM
Created by:

Charge number:1 Tolerance:2.00(mmu) Unsaturation Number:5.0 .. 20.0 (Fraction:Both)
Element:^{12}C:0 .. 22, ^{1}H:1 .. 50, ^{35}Cl:1 .. 1, ^{37}Cl:0 .. 1, ^{14}N:0 .. 3, ^{16}O:3 .. 5

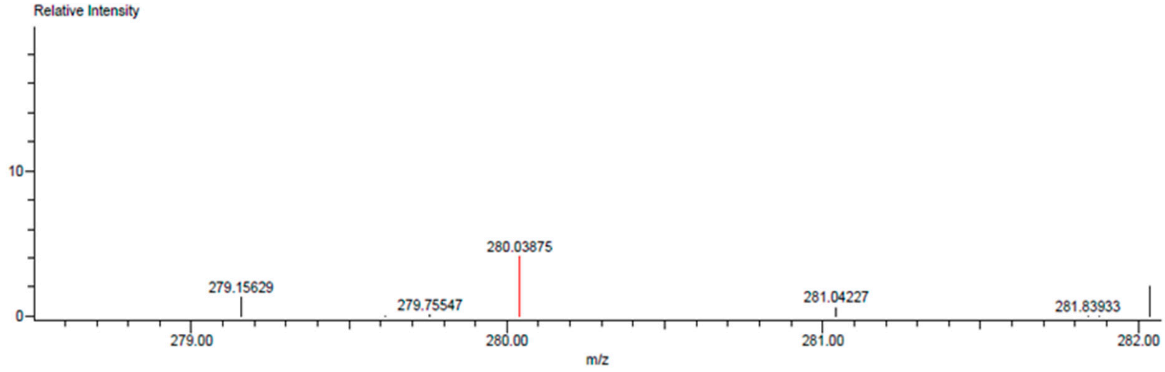

Mass	Intensity	Calc. Mass	Mass Difference (mmu)	Mass Difference (ppm)	Possible Formula	Unsaturation Number
280.03875	6898.75	280.03766	1.09	3.88	$^{12}C_{13}{}^{1}H_{11}{}^{35}Cl_{1}{}^{14}N_{1}{}^{16}O_{4}$	8.5

(4c)

Data:U-280 NCLM-MS
Sample Name:Lizbeth Rodriguez
Description:
Ionization Mode:ESI+
History:Determine m/z[Peak Detect[Centroid,30,Area];Correct Base[1.0%]];Correct Base[5.0%];Average(MS[1] 1..1)

Acquired:2/28/2018 10:32:39 AM
Operator:AccuTOF
Mass Calibration data:Cal Peg 600
Created:3/7/2018 9:48:45 AM
Created by:

Charge number:1
Tolerance:2.00(mmu)
Unsaturation Number:5.0 .. 20.0 (Fraction:Both)
Element:${}^{12}C$:0 .. 22, ${}^{1}H$:1 .. 50, ${}^{35}Cl$:1 .. 1, ${}^{37}Cl$:0 .. 1, ${}^{14}N$:0 .. 3, ${}^{16}O$:3 .. 5, ${}^{32}S$:1 .. 1

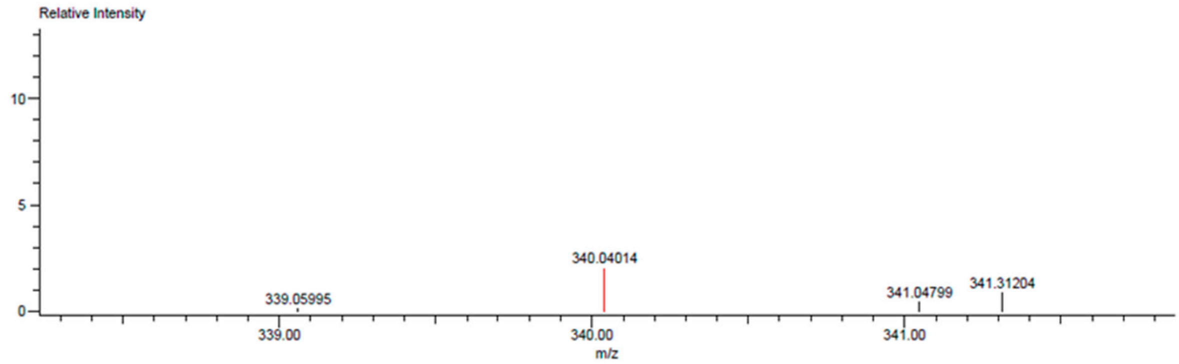

Mass	Intensity	Calc. Mass	Mass Difference (mmu)	Mass Difference (ppm)	Possible Formula	Unsaturation Number
340.04014	41284.85	340.04103	-0.89	-2.61	${}^{12}C_{15}{}^{1}H_{15}{}^{35}Cl_{1}{}^{14}N_{1}{}^{16}O_{4}{}^{32}S_{1}$	9.5

(4e)

**Data Filename**	NCL2Asn.d	**Sample Name**	NCl2Asn
**Sample Type**	Sample	**Position**	P1-B5
**Instrument Name**	LC QTOF-LANCIC	**User Name**	
**Acq Method**	Ine directa pos.m	**Acquired Time**	8/2/2018 2:34:02 PM
**IRM Calibration Status**	Success	**DA Method**	Default.m
**Comment**			

**Sample Group**		**Info.**	
**Stream Name**	LC 1	**Acquisition SW Version**	6200 series TOF/6500 series Q-TOF B.06.01 (B6172 SP1)

User Spectra

Fragmentor Voltage	Collision Energy	Ionization Mode
150	0	ESI

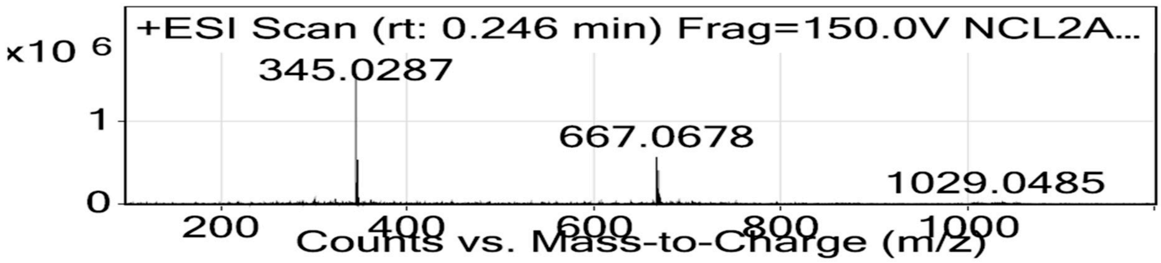

Supplement: Supplementary file 1 [file molecules-24-04285-s001.pdf]
